# Chinese herbal formula in the treatment of metabolic dysfunction-associated steatotic liver disease: current evidence and practice

**DOI:** 10.3389/fmed.2024.1476419

**Published:** 2024-10-08

**Authors:** Shao-Hong Tao, Yu-Qing Lei, Yi-Mei Tan, Yu-Bo Yang, Wei-Ning Xie

**Affiliations:** ^1^Affiliated Guangdong Hospital of Integrated Traditional Chinese and Western Medicine of Guangzhou University of Chinese Medicine, Guangzhou University of Chinese Medicine, Foshan, Guangdong, China; ^2^School of Traditional Chinese Medicine, Jinan University, Guangzhou, Guangdong, China; ^3^Department of Scientific Research, Guangdong Provincial Hospital of Integrated Traditional Chinese and Western Medicine, Foshan, Guangdong, China

**Keywords:** metabolic dysfunction-associated steatotic liver disease, Chinese herbal formula, traditional Chinese medicine, mechanisms, review

## Abstract

Metabolic dysfunction-associated steatotic liver disease (MASLD), formerly known as nonalcoholic fatty liver disease, continues to rise with rapid economic development and poses significant challenges to human health. No effective drugs are clinically approved. MASLD is regarded as a multifaceted pathological process encompassing aberrant lipid metabolism, insulin resistance, inflammation, gut microbiota imbalance, apoptosis, fibrosis, and cirrhosis. In recent decades, herbal medicines have gained increasing attention as potential therapeutic agents for the prevention and treatment of MASLD, due to their good tolerance, high efficacy, and low toxicity. In this review, we summarize the pathological mechanisms of MASLD; emphasis is placed on the anti-MASLD mechanisms of Chinese herbal formula (CHF), especially their effects on improving lipid metabolism, inflammation, intestinal flora, and fibrosis. Our goal is to better understand the pharmacological mechanisms of CHF to inform research on the development of new drugs for the treatment of MASLD.

## 1 Introduction

With the rapid growth of economy and significant changes in lifestyle, metabolic dysfunction-associated steatotic liver disease (MASLD), formerly known as nonalcoholic fatty liver disease (NAFLD), is becoming increasingly prevalent (1). The global prevalence of MASLD is approximately 30% and appears to be increasing (2). MASLD was recently proposed to replace the term “NAFLD” in the Delphi consensus statement (3). MASLD goes beyond the limitations of the previous nomenclature of NAFLD. First, the new nomenclature removes the stigmatizing adjectives “non-alcoholic” and “fatty.” Second, the adjective “metabolic” has been added, which emphasizes the impact of the underlying metabolic pathophysiology of liver disease on cardiac metabolism. Zhou et al. showed that the prevalence and severity of NAFLD and MASLD remain similar (4) and previous relevant literature remains valid (5). MASLD comprises a range of liver damage, from metabolic-dysfunction-associated steatotic liver (MASL—hepatic steatosis without transaminitis) to metabolic-associated steatohepatitis (MASH), liver fibrosis, and ultimately may lead to hepatocellular carcinoma (6). Moreover, the correlation between MASLD and other chronic diseases, such as chronic liver disease, cardiovascular disease, endocrinopathies, and chronic kidney disease, has been reported (7–10). In conclusion, MASLD causes enormous clinical and economic burdens and is one of the leading causes of death.

Currently, there is no officially approved clinical drug for the management of MASLD (11). For one thing, the main treatment methods for MASLD are changing lifestyles, controlling diet, and strengthening exercise to improve liver steatosis and inflammation (12). However, lifestyle modification is hard to achieve weight loss goals and maintain for a long time for most patients. Limitations such as cost, potential side effects, and patient acceptance of invasive bariatric surgery should also be fully considered. For the other thing, numerous drugs have been discarded after unsuccessful clinical trials. A single mechanism has been emphasized in current drug research, while the complex pathophysiology of MASLD has been neglected. Preliminary results of single-drug trials showed that inflammation improved in less than 50% of MASH patients (13). A multitude of novel pharmaceutical agents are undergoing various stages of development but have not yet been formally approved for use, including antidiabetic drugs, peroxisome proliferator-activator receptor modulators, farnesoid X receptor agonists, and fibroblast growth factor analogs (13). Accordingly, the creation of pharmaceuticals for treating MASLD represents a significant unmet medical need. It is necessary and urgent to find economical, safe, and efficient drugs for the treatment of MASLD.

Traditional Chinese medicine has been used for over 2,000 years to treat a wide range of diseases, including those related to the liver. Many traditional Chinese medicines have great potential for preventing and treating MASLD, such as *H. erinaceus*, which treats a variety of gastrointestinal disorders by modulating intestinal microbiota and improving inflammation (14). CHF contains a wide variety of traditional Chinese medicines, which are a major source of herbal products and natural medicines, and are an important resource for the production of hepatoprotective drugs. CHF contains complex chemical components that can treat diseases through multi-target multi-pathway and multi-level pharmacological activity (15, 16). Therefore, CHF may be a promising candidate for solving the limitations of current single-target drug therapy strategies. In recent years, progress has been made in the development of CHF for MASLD. This review presents an overview of the underlying pathomechanisms of MASLD. Moreover, in light of the latest findings from basic and clinical research, we present a summary of the anti-MASLD mechanisms of CHF, with a particular emphasis on their specific effects on lipid metabolism, intestinal flora, liver inflammation, and fibrosis.

## 2 The pathogenesis of MASLD

**Figure 1:**
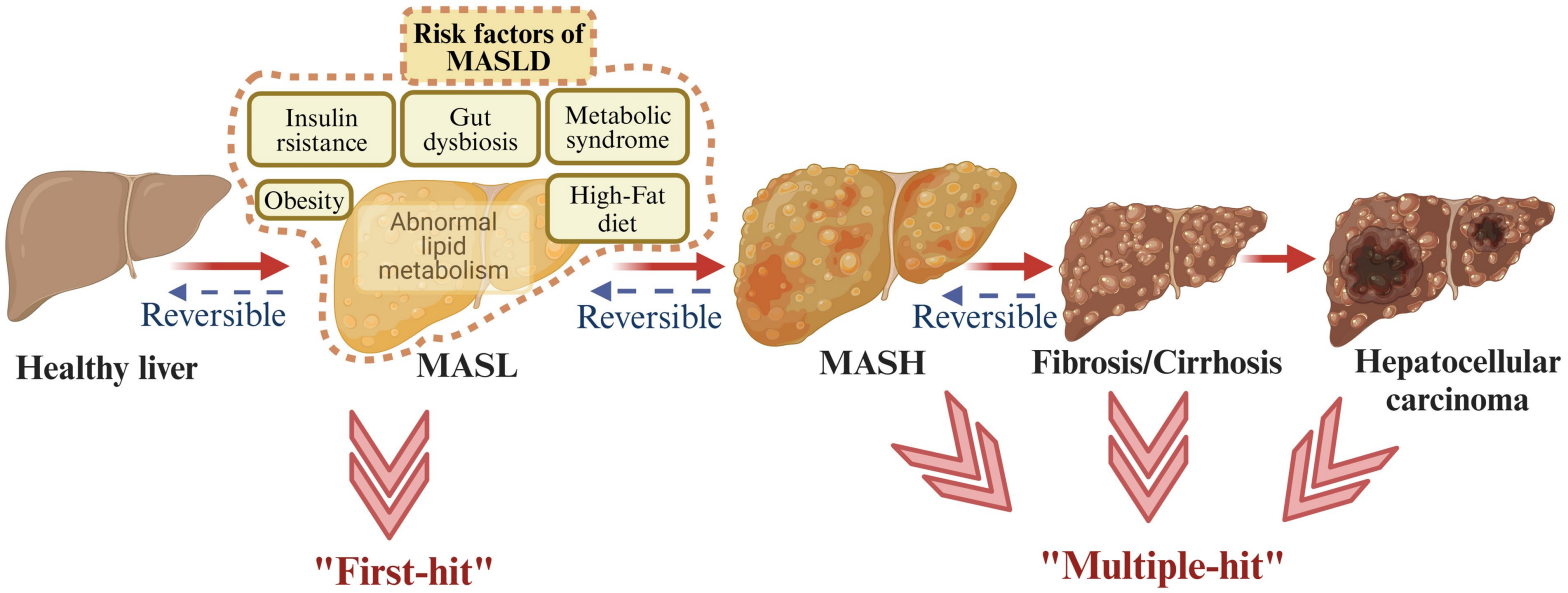
Metabolic dysfunction-associated steatotic liver disease (MASLD) spectrum. MASLD involves four different stages, starting from MASL, the appearance of MASH, the development of fibrosis, and ultimately possibly leading to hepatocellular carcinoma. The ‘two-hit’ theory and the ‘multiple-hit’ hypothesis have been proposed to describe the pathogenesis of MASLD (created with BioRender.com).

MASLD comprises a range of liver damage, from MASL to MASH, liver fibrosis, and ultimately may lead to hepatocellular carcinoma (6). In order to characterize the pathogenesis of MASLD, the ‘two-hit’ theory and the ‘multiple-hit’ hypothesis have been proposed. The ‘two-hit’ theory proposed that steatosis and oxidative stress play important roles in MASLD progression (17). The ‘multiple-hit’ hypothesis involved richer and more accurate factors, such as abnormal lipid metabolism, insulin resistance (IR), inflammation, gut microbiota imbalance, fibrosis, cirrhosis, and so on (18) (Figure 1).

### 2.1 IR and abnormal lipid metabolism

**Figure 2:**
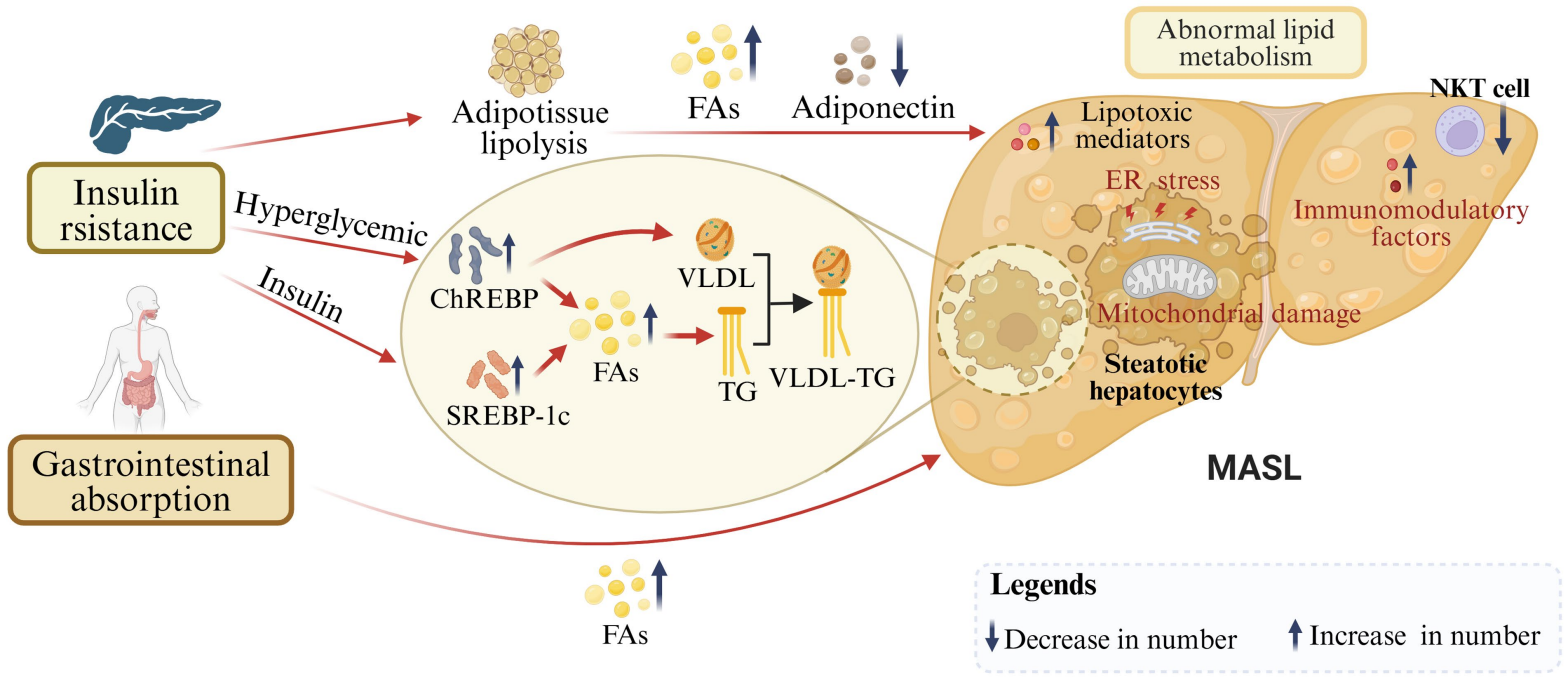
Insulin resistance and abnormal lipid metabolism in the progression of MASLD. On the one hand, under IR conditions, SREBP-1c and ChREBP are activated to further regulate derived adipogenesis and FAs production. On the other hand, dietary-derived FAs are over-accumulated in hepatocytes. In addition, VLDL-TG molecules are oversized, leading to reduced lipid transport. With excessive FAs, production of lipotoxic mediators, and reduction of adiponectin, steatosis, endoplasmic reticulum (ER) stress, mitochondrial damage, and inflammation occur in hepatocytes (created with BioRender.com).

MASLD shares its key risk factor of IR with increasing metabolic problems of type 2 diabetes mellitus, metabolic syndrome, obesity, hypertension, and dyslipidemia (19). On the one hand, IR causes low glucose disposal in adipose and muscle tissue, and further excessive delivery of fatty acids (FAs) to the liver (20). Derived lipogenesis (DNL) represents a metabolic process whereby non-lipids (predominantly carbohydrates) are converted into FAs, which constitute an essential component of lipid metabolism (21). The precise mechanisms underlying DNL in MASLD remain unclear, but sterol regulatory element-binding protein 1c (SREBP-1c) and carbohydrateresponsive element binding protein (ChREBP) have been believed to be coregulators (22). SREBP-1c is activated by insulin and controls enzyme activation in DNL. SREBP1c has been reported to be highly expressed in MASLD patients, which is associated with insulin resistance (23). In hyperglycemic and postprandial states, ChREBP is activated, increasing enzyme activity and gene transcription in the DNL pathway (24). Elevated levels of ChREBP have been demonstrated in the liver biopsies of patients with MASH. Increased ChREBP has beneficial effects on both lipid and glucose metabolism by separating hepatic steatosis from insulin resistance (24). ChREBP also regulates the synthesis of very-low-density lipoprotein (VLDL), which facilitates the transport of triglycerides (TG) in hepatocytes (23). In patients with MASLD, VLDL synthesis and secretion exhibit an increase, yet stabilization occurs when hepatic lipid accumulation exceeds 10% (25). In addition, VLDL-TG molecules in obese individuals are so large that they are unable to penetrate the hepatic vascular sinus and end up in the bloodstream, which is one of the reasons why lipids accumulate in the liver (25). On the other hand, the excessive accumulation in hepatocytes of fat originating from diet leads to abnormal lipid uptake, synthesis, oxidation, and output in the liver (26). With free FAs overloaded, steatosis occurs and lipotoxic mediators comprising diacylglycerols, saturated free FAs, ceramide, and sphingolipids are produced, which further causes endoplasmic reticulum (ER) stress and mitochondrial damage in hepatocytes (27). Meanwhile, the reduction of adiponectin leads to an increase in FAs synthesis and enhancement of mitochondrial β-oxidation (28). Furthermore, IR is always associated with inflammation and lots of immunomodulatory factors, including IL-1, IL-6, TNFα, monocyte chemoattractant protein-1, and the IκB kinase β/nuclear factor-κB (NF-κB) pathway (29, 30). In conclusion, IR is closely associated with steatosis and inflammation which are important factors in the progression of MASLD (Figure 2).

### 2.2 Immunological mechanisms

**Figure 3:**
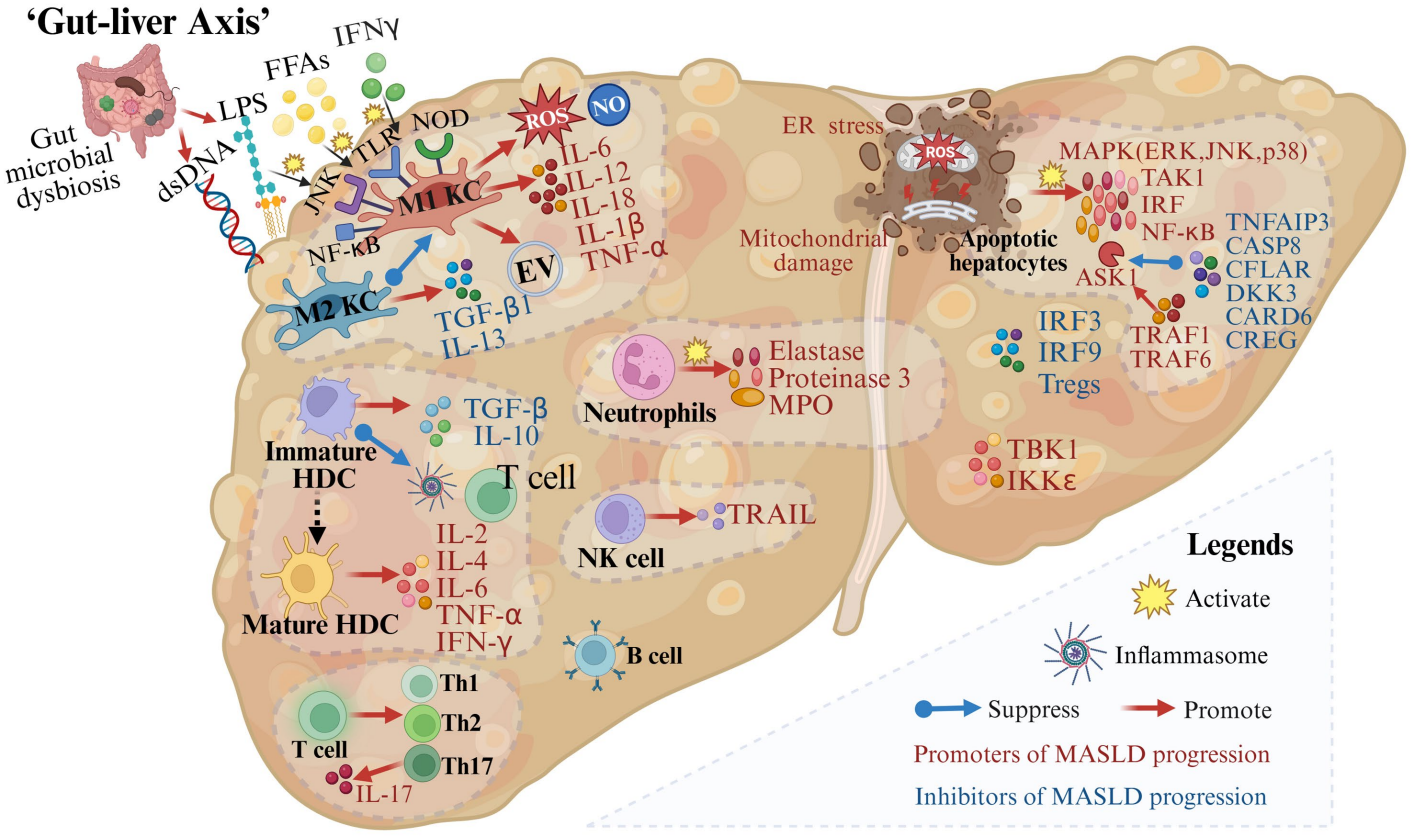
Gut microbial dysbiosis and immune mechanisms in the progression of metabolic dysfunction-associated steatotic liver disease. A variety of immune cells and immune factors are involved in the progression of MASLD. In addition, in the case of impaired intestinal permeability and intestinal microbial disorders, immune cells are activated and produce inflammation-related molecules (created with BioRender.com).

Inflammation is a key driver in the progression of MASLD. The immune response is critical for tissue repair, yet excessive immune activation may cause liver tissue damage (Figure 3).

#### 2.2.1 Innate immune system

During MASLD, M1 Kupffer cells (KCs) are induced by mediators such as circulating FFAs, interferon-γ (IFN-γ), and lipopolysaccharide (LPS) through pattern recognition receptors including membrane-bound toll-like receptors (TLRs) and cytoplasmic nucleotide oligomerization domain-like receptors (31–33). Then activated M1 KCs secrete extracellular vesicles and pro-inflammatory cytokines such as IL-6, IL-1β, and TNF-α (34–37). On the contrary, M2 KCs decrease the progression of MASLD by inducing M1 KCs apoptosis and releasing transforming growth factor (TGF)-β1 and IL-13 which can promote liver remodeling and tissue repair (38, 39). Therefore, the balance between M1 KCs and M2 KCs is crucial for liver homeostasis.

Hepatic dendritic cells (HDCs), important immune cells in the liver, play a dual role in MASLD development and progression. In the state of immune tolerance, immature HDCs secrete TGF-β and IL-10, suppress inflammasome activation, restrict T cell expansion, and remove necrotic debris and apoptotic cells to maintain liver homeostasis (40, 41). Conversely, when HDCs are transformed into an active state, their mature forms produce inflammatory cytokines such as IL-2, IL-4, IL-6, TNF-α, and IFN-γ, which further lead to pro-inflammatory responses (41).

Other innate immune cells are also associated with the progression of MASLD. It is reported that neutrophils and factors released from neutrophils including elastase, proteinase 3, and myeloperoxidase are associated with inflammation and fibrosis in MASLD patients (42–44). The number of NKT cells decreases during steatosis and increases in the middle and late stages of the disease. NK cells may promote hepatocyte apoptosis, inflammation, and fibrosis by secreting IFN-γ and TRAIL (45, 46).

In addition, immune factors are indispensable components of the progression of MASLD. Following tissue damage, members of the major mitogen-activated protein kinase (MAPK) families including the extracellular signal-regulated kinase (ERK), Jun N-terminal kinase (JNK), and p38 are activated (47). Besides, upstream and downstream effector kinases of MAPK including TGFβ-activated kinase 1 (TAK1), apoptosis signal-regulating kinase 1 (ASK1), interferon regulatory factors (IRF) and NF-κB are also involved in regulating the inflammatory response in hepatocytes (47). The activation of ASK1, a crucial mechanism in the progression of MASLD, is promoted by tumor necrosis factor receptor-associated factor 1 (TRAF1) (48–50) and TRAF 6 (51), while it is suppressed by deubiquitinase TNFα-induced protein 3 (52), CASP8 and Fadd-like apoptosis regulator (53), dickkopf-3 (54), caspase recruitment domain 6 (55) and cellular repressor of E1A-stimulated genes (56). TANK-binding 1 kinase (57) and IKKε (58) promote hepatocyte steatosis. In contrast, tumor necrosis factor receptor-associated factor 5 has been reported to alleviate hepatic steatosis (20), IRF3 to alleviate IR and reduce hepatic lipid accumulation (59), as well as IRF9 to reduce steatosis and inflammation (60–62).

#### 2.2.2 Adaptive immune system in MASLD

Increased recruitment of CD4+ T cells and CD8+ T cells has been reported in the liver of patients diagnosed with MASH (63–65). Stimulated by inflammation, CD4+ T-cells differentiate into Th1, Th2, and Th17 populations and produce specific cytokines (66). IL-17, one of these cytokines, interferes with the insulin signaling pathway and activates hepatic stellate cells (HSCs), leading to the progression of inflammation and fibrosis (67, 68). Tregs, one of the T-cell subsets, have been reported to have reduced levels in the liver and circulation of MASLD animal models and patients (69). Increasing the number of Tregs reduces liver inflammation and injury in mice (70).

The role of B cells in the pathogenesis of MASLD is not fully understood. IgA has been reported to be associated with hepatocellular inflammation, fibrosis, and carcinoma, and it can predict the progression of advanced liver disease (71–74).

### 2.3 Gut-liver axis

With impaired intestinal permeability and gut microbial dysbiosis, plentiful gut-derived products (such as LPS and dsDNA) or the microbiota enter the portal circulation and activate immune cells through signaling pathways such as TLR9, TLR4, NF-κB, and JNK (75–77). Activated immune cells promote the secretion of cytokines such as TNF-α, IL-1β, IL-6, IL-12, and IL-18 as well as the production of inflammation-related molecules such as ROS and NO, leading to inflammation in hepatocytes (75–77) (Figure 3). “Fonte Essenziale” water, a bicarbonate-sulfate-calcium–magnesium water, has been shown to produce beneficial effects on the liver-gut axis, modulating gut microbiota and gastrointestinal hormones, and further improving functional gastrointestinal symptoms in patients with MASLD (78, 79).

### 2.4 Apoptosis and fibrosis

**Figure 4:**
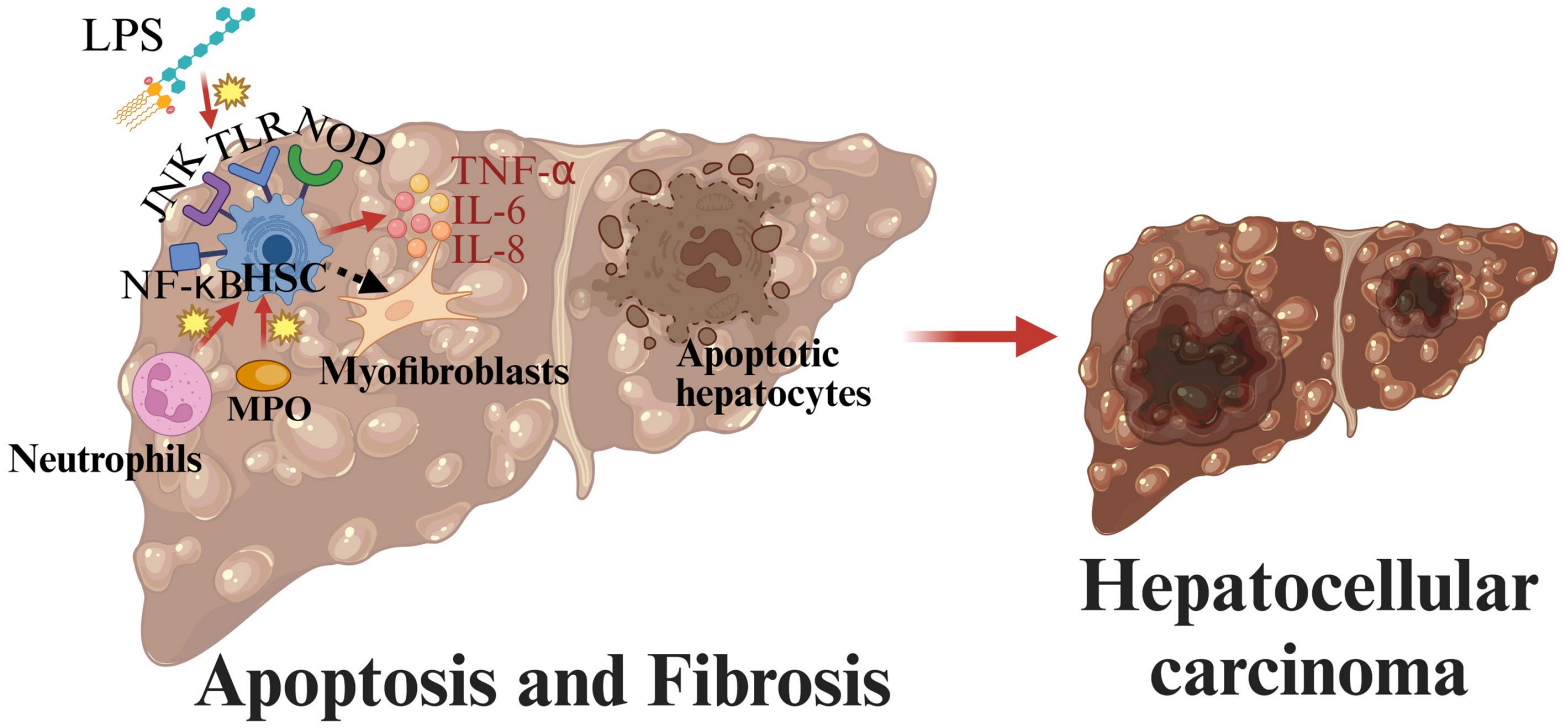
Pathogenesis of MASLD from apoptosis and fibrosis to hepatocellular carcinoma. In MASLD, hepatic stellate cells (HSCs) are the key to the process of fibrogenesis. Activated HSCs are converted to myofibroblasts. Unregulated structural remodeling and fibrogenesis may lead to cirrhosis and hepatocellular carcinoma (created with BioRender.com).

Apoptosis and fibrosis are also important for MASLD progression. Apoptosis occurs throughout the course of MASLD, and it is closely associated with inflammation and fibrosis (80). Hepatocyte damage and death are caused by FFA, lipotoxicity, intestinal microbial products, and inflammasome activation (80). Following hepatocyte injury and apoptosis, major MAPK family members along with their upstream and downstream effector kinases are activated and involved in regulating inflammation (47). Moreover, in MASLD, hepatic stellate cells (HSCs) are the key to the process of fibrogenesis (39). HSCs are activated by LPS through TLR4 which promotes the production and release of cytokines such as TNF-α, IL-6, and IL-8, leading to the activation of signal pathways such as JNK and NF-κB (81). In addition, myeloperoxidase and neutrophils can also lead to the activation of HSCs (44, 82). Activated HSCs are converted to myofibroblasts (83). Unregulated structural remodeling and fibrogenesis may lead to cirrhosis and hepatocellular carcinoma (84) (Figure 4).

## 3 The mechanisms of CHF for treating MASLD

CHF has been utilized for the treatment of liver disease for over two millennia, making a significant contribution to the field of healthcare. Although considerable progress has been made in the study of the pathogenesis of MASLD, no effective drugs have been approved for clinical use. Fortunately, CHF may be a potential treasure trove for the treatment of MASLD. To explore the mechanism of the therapeutic effect of CHF on MASLD, many animal experiments have been carried out. We list recent promising CHFs for the treatment of MASLD and their therapeutic mechanisms (Table 1).

**Table 1:** List of Chinese herbal formulas with potential therapeutic benefits for MASLD.

Chinese herbal formula	Component	Model	Reported targets	Associated mechanism of action					Duration	Published time
				Lipid metabolism	Inflammation	Gut microbiota	Fibrosis	Others		
Hedansanqi Tiaozhi Tang (89)	*Salvia miltiorrhiza* Bge. *Crataegus pinnatifida* Bge. *Panax notoginseng* (Burk.) F. H. Chen *Nelumbo nucifera* Gaertn.	Male SD rats (HFD); 3T3-L1 murine embryo fibroblast cells	↑ 3T3-L1 adipocytes, Nrf2/HO-1	+	+			↓ Oxidative stress	4 weeks	2020
Si-Wei-Qing-Gan-Tang (90)	*Artemisia capillaris* Thunb. *Oldenlandia diffusa* (Willd.) Roxb. *Gardenia jasminoides* J. Ellis *Taxillus sutchuenensis* (Lecomte) Danser	Male SD rats (MCDD)	↓ ERK1/2, p38 MAPK	+	+			↑ Autophagy	4 weeks	2020
Jiang Zhi Granule (97)	*Salvia miltiorrhiza* Bge. *Folium nelumbinis* *Polygala tenuifolia* Willd. *Artemisia capillaris* Thunb. *Gynostemma pentaphyllum* (Thunb.) Makino	Male SD rats (HFD)	↓ TLR-44, MyD88; naive CD4+ T cells into Th1 cells	+	+			↓ Oxidative stress, protect immunological barrier of intestinal mucosa	4 weeks	2021
Si Miao Formula (99)	*Phellodendron chinense* C. K. Schneid. *Atractylodes lancea* (Thunb.) DC. *Achyranthes bidentata* Blume *Coix lacryma-jobi L*. var. *mayuen*	Male C57BL/6 mouse (High fat/high sucrose diet)	↓ Acly, FAS, ACC, Scd-1, IL-1β, Nlrp-3	+	+	+		↓ IR	16 weeks	2021
Qushihuayu formula (103)	*Curcuma longa* L. *Artemisia capillaris* Thunb. *Gardenia jasminoides* J. Ellis *Hypericum japonicum* Thunb. ex Murray *Polygonum cuspidatum* Sieb. et Zucc.	Male Wistar rats (MCD); male Wistar rats (MCDD)	↑ PPAR-γ and phosphorylated p65 translocating into nucleus, HSCs reprogramming. ↓MAPK	+			+		8 weeks	2021
Zexie–Baizhu Decoction (85)	*Alisma plantago-aquatica* L. *Atractylodes macrocephala* Koidz.	Male C57/BL6 mouse (Gubra-amylin MASH diet)	↑ Sirt1, AMPK	+				↑ Autophagy	12 weeks	2022
Livsooth authentic herbal formula (87)	*Pueraria lobata* (Willd.) Ohwi *Pueraria montana* (Lour.) Merr. *Hovenia dulcis* Thunb. *Lonicera japonica* Thunb. *Siraitia grosvenorii* (Swingle) C. Jeffrey ex A. M. Lu & Zhi Y. Zhang	Male C57BL/6 mouse (HFD)	↑ AMPK/ACC hepatic antioxidant enzymes activities, β-oxidation. ↓FAS, SREBP1	+				↓ Oxidative stress, hyperglycemia	18 weeks	2022
Danshao Shugan Granule (88)	*Salvia miltiorrhiza* Bge. *Paeonia veitchii* Lynch *Bupleurum komarovianum* Lincz. *Curcuma aromatica* Salisb. *Cyperus rotundus* L., et al.	Male SD rats (HFD)	↓ NF-κB, malondialdehyde values. ↑ Superoxide dismutase activity.	+	+				8 weeks	2022
Quzhi Formula (92)	*Polygonum cuspidatum* Sieb. et Zucc. *Cassia obtusifolia* L. *Crataegus pinnatifida* Bge.	Male C57BL/6 SPF mouse (choline-deficient, l-amino acid-defined, and HFD); mouse hepatocyte (FFA)	↓ Bip/eIF2α	+	+			↓ ER stress	4 weeks	2022
Yinchenhao Tang (93)	*Artemisia capillaris* Thunb. *Gardenia jasminoides* J. Ellis *Rheum Palmatum* L.	Male Kunming mouse (HFD)	↑ NR1H4, APOA1	+	+				4 weeks	2022
Shuangyu Tiaozhi decoction (95)	*Dioscorea oppositifolia* L. *Dioscorea septemloba* Thunb.	Male SD rats (HFD); High-fat HepG2 cells (FFAs)	↑ Relative mRNA and protein levels of ESR1 and p-GSK-3β. ↓ Relative mRNA and protein levels of mTOR, FASN, HIF-1α, and VEGFA	+	+			↓ IR	8 weeks	2022
Lingguizhugan decoction (96)	*Poria cocos* (Schw.) Wolf *Cinnamomum cassia* Presl *Atractylodes macrocephala* Koidz. *Glycyrrhiza uralensis* Fisch.	Male C57BL/6 J mouse (HFD) bone-marrow-derived macrophages and primary liver macrophages	↓ STING-TBK1-NF-κB	+	+			↓ Oxidative stress, hepatic mitochondrial damage, mitochondrial DNA release	9 weeks	2022
Tianhuang formula (101)	*Panax notoginseng* (Burk.) F. H. Chen *Coptis chinensis* Franch.	Male C57BL/6 J Narl mouse (HFD)	↑ Lactobacillus-5-Methoxyindoleacetate-Nrf2.			+		↓ Oxidative stress	6 weeks	2022
Pien Tze Huang (104)	*Moschus berezovskii* Flerov *Calculus bovis* snake gall *Panax notoginseng* (Burk.) F. H. Chen	Male C57BL/6 mouse (HFD and MCDD)	↓ NF-Κb, IκBα	+	+		+		2 weeks	2022
Si-Ni-San (86)	*Bupleurum falcatum* L. *Citrus aurantium* L. *Paeonia lactiflora* Pall. *Glycyrrhiza aspera* Pall.	HFD-fed C57BL/6 mouse; oleic acid-induced HepG2 cells	↓ Lipid droplet, YAP1, PLIN2	+					2 weeks	2023
Erchen Decoction (100)	*Pinellia ternata* (Thunb.) Breit. *Poria cocos* (Schw.) Wolf *Glycyrrhiza uralensis* Fisch. *Zingiber officinale* Roscoe *Citrus reticulata* Blanco, et al.	Male SD rats (HFD)	↑ Gut microbiota-drived butyric acid contents, FAO, H3K9ac; ↓ HDAC1	+		+			4 weeks	2023
Qing-Zhi-Tiao-Gan-Tang (102)	*Bupleurum scorzonerifolium* Willd. *Citrus aurantium* L. *Paeonia lactiflora* Pall. *Coptis chinensis* Franch. *Cassia obtusifolia* L., et al.	Male C57BL/6 mouse (MCDD)	↓ Col1a1, TGF-β, Tnf-α, IL-6, IL-1β	+	+		+	↑ FAs degradation, bile secretion, and steroid biosynthesis. ↓fibrosis genes	5 weeks	2023
Chaihu Shugan powder (91)	*Bupleurum scorzonerifolium* Willd. *Ligusticum chuanxiong* Hort. *Citrus aurantium* L. *Citrus reticulata* Blanco *Paeonia lactiflora* Pall., et al.	male Wistar rats (HFD)	↓ MiRNAs; gene and protein levels of ACACA, FASN, and other FAs biosynthesis-related enzymes	+	+			↑ Liver surface microcirculation	8 weeks	2024
Scutellaria-coptis herb couple (94)	*Scutellaria baicalensis* Georgi *Coptis chinensis* Franch.	Male SD rats (HFD); HepG2 and RAW264.7 cells	↑ Nrf2 and FXR	+	+			↓ Oxidative stress	22 days	2024
Si Miao Formula (98)	*Phellodendron chinense* C. K. Schneid. *Atractylodes lancea* (Thunb.) DC. *Achyranthes bidentata* Blume *Coix lacryma-jobi L*. *var*. *mayuen*	Male C57BL/6 J mouse (High fat/high sucrose)	↓ FASN, CPT1A, CPT2, CD36, IL-6, IL-1β, Tnf-α	+	+				16 weeks	2024

↑ promote; ↓ inhibit.

### 3.1 Improving lipid metabolism

The ‘Pandora’s Box’ of MASLD is opened by abnormal lipid metabolism and excessive transportation of FFAs to the liver. Many CHFs can treat MASLD by regulating lipid metabolism. Zexie–Baizhu Decoction (85) activates AMPK and Sirt1 pathway, and then improves lipid metabolism by suppressing gluconeogenesis, inhibiting lipogenesis, activating fatty utilization, promoting FAs oxidation, prompting autophagy, and increasing bile acid metabolism. Si-Ni-San (86) reduces Yes-associated protein 1 in hepatocytes, further reducing lipid droplet deposition. Livsooth authentic herbal formula (87) regulates glucose and lipid metabolism in high fat diet-fed induced mice by promoting β-oxidation, increasing AMPK/ACC activation, and downgrading FAS and SREBP1.

### 3.2 Ameliorating inflammation

Inflammation within the liver is an important driver of MASLD development. Many CHFs can both improve lipid metabolism and inflammation by acting on different targets. Wang et al. found that compared with the negative control group, the positive therapeutic effect of Danshao Shugan Granule (88) on hepatic steatosis and inflammation in MASLD rats was related to the decrease of NF-κB expression, the reduction of malondialdehyde, and the increase of superoxide dismutase activity. Hedansanqi Tiaozhi Tang (89) exerts lipolysis-promoting and liver-protecting effects by enhancing the nuclear factor-erythroid 2-related factor 2 (Nrf2)/Heme oxygenase 1 antioxidant pathway in hepatocytes and the antioxidant activity of 3T3-L1 adipocytes. Si-Wei-Qing-Gan-Tang (90) ameliorates MASH in rat models by activating autophagy and downregulating NF-κB through p38 MAPK and ERK1/2 signals. Chaihu Shugan powder (91) can reduce FAs synthesis, improve hepatic surface microcirculation disorders, and ultimately reduce transaminase and serum lipid levels by reversing the high expression of 15 miRNAs. Quzhi formula (92) is involved in multi-anti-MASH mechanisms, including inhibition of ER stress, lipid accumulation, and inflammation through Bip/eIF2α Signaling. Yinchenhao Tang (93) has been demonstrated to markedly improve lipid metabolism, reduce body weight, and diminish AST and ALT levels in mice, which is associated with increased NR1H4 and APOA1 expression. The Scutellaria-coptis herb couple (94) alleviates MASH by inhibiting lipotoxicity, inflammation, and oxidative stress via activating the NRF2 and FXR Signaling. The protective effect of Shuangyu Tiaozhi decoction (95) against MASLD is based on the molecular mechanism of the relative mRNA and protein levels of ESR1, p-GSK-3β, mTOR, FASN, HIF-1α, and VEGFA, which ameliorate lipid deposition, inflammation, and IR. The Lingguizhugan decoction (96) alleviates hepatic lipid deposition by suppressing STING-TBK1-NF-κB single in hepatic macrophages.

### 3.3 Modulating gut microbiota

Dysregulated gut microflora can activate immune cells and promote the progression of MASL to hepatitis and fibrosis. Some CHFs target the ‘liver-gut axis’ to regulate gut microbiota and reduce intestinal mucosal damage to curb the development of MASLD. Jiang Zhi Granule (97) protects intestinal mucosal immune barrier in MASH rats by inhibiting the TLR-4/MyD88 signaling pathway. Si Miao Formula inhibits the expression of inflammatory factors, suppresses the production and transport of FAs (98, 99), and modulates the composition of the gut microbiota, especially increasing the abundance of *Akkermansia muciniphila* (99). The results also indicate that compared to fenofibrate, Si Miao Formula (99) has a more significant effect on TG reduction. Erchen Decoction (100) and Tianhuang formula (101) are also utilized to treat MASLD by modulating intestinal microbial flora diversity.

### 3.4 Alleviating fibrosis

The occurrence of fibrosis plays a sufficient role in the progression of MASLD. Chu et al. predicted by transcriptome-based multi-scale network pharmacology logical platform and then proved through animal experiments that Qing-Zhi-Tiao-Gan-Tang (102) inhibits the expression of fibrosis genes (such as *TGF-β, Col1a1*), reduces the levels of inflammatory factors (such as IL-1β, IL-6, and Tnf-α), and improves the ‘steroid biosynthesis’, ‘bile secretion’ and ‘FAs degradation’ pathways. Qushihuayu formula (103) may exert hepatoprotective effects by enhancing the reprogramming of HSCs, promoting the translocation of p-p65 and PPAR-γ to the nucleus, inhibiting the phosphorylation of MAPK pathway, and further alleviating steatosis and fibrosis. Pien Tze Huang (104) holds a beneficial role in steatosis, inflammation, and fibrosis of MASLD by inhibiting the NF-κB pathway and the degradation of inhibitor of κBα.

In conclusion, it has been demonstrated that CHF, which contains complex chemical components, plays a beneficial role in improving lipid metabolism, ameliorating inflammation, modulating gut microbiota, and inhibiting hepatic fibrosis through multi-target, multi-pathway, and multi-level pharmacological mechanisms. Therefore, CHF may be a promising candidate to overcome the limitations of the current single-target drug treatment strategy.

## 4 Clinical practices of CHF on MASLD

A number of scholars have conducted clinical trials to explore the clinical effectiveness and safety of CHF in MASLD, and they have confirmed the multiple therapeutic effects and clinical application potential of CHF. Randomized controlled trials (RCTs) comparing CHFs with placebo or other pharmaceutical agents have been explored and the results are encouraging (Table 2).

**Table 2:** Clinical trials of Chinese herbal formulas for the treatment of MASLD.

Chinese herbal formula	Component	Test group (sample size, male: female)	Control group (sample size, male: female)	Assessment of hepatic steatosis	Outcome	Adverse events (number of cases)	Published time
Shugan Xiaozhi Decoction (108)	*Artemisia capillaris* Thunb. *Gardenia jasminoides* Ellis *Bupleurum scorzonerifolium* Willd. *Paeonia lactiflora* Pall. *Citrus aurantium* L., et al.	CHF (40, 29:11)	Polyene phosphatidylcholine capsules (40, 27:11)	FibroScan, B-ultrasound	↓ CAP, E, TC, TG, ALT, AST, BMI, fasting blood glucose	Diarrhea (7)	2018
Qinjiang Baoling Decoction (105)	*Bupleurum scorzonerifolium* Willd. *Paeonia lactiflora* Pall. *Schisandra chinensis* (Turcz.) Baill. *Poria cocos* (Schw.) Wolf *Curcuma phaeocaulis* Val., et al.	CHF + lifestyle and symptomatic treatment (50, 28:22)	Diet, exercise, and symptomatic treatment (50, 31:19)	B-mode ultrasound, liver/spleen CT ratio	↓ TC, ALT, GGT, TCM syndrome integral	/	2019
Qinghua Decoction (109)	*Nelumbo nucifera* Gaertn. *Atractylodes macrocephala* Koidz. *Sedum sarmentosum* Bunge *Poria cocos* (Schw.) Wolf *Salvia miltiorrhiza* Bge., et al.	CHF + lifestyle (36, 24:12)	Qianggan Capsule+lifestyle (36, 21:15)	Fibroscan, B-ultrasound	↑ Total effective rate ↓CAP, TG, ALT, AST, GGT	NR	2019
Zhibitai Capsule (112)	*Crataegus pinnatifida* Bge. *Atractylodes macrocephala* Koidz. *Alisma plantago-aquatica* L., et al.	CHF + Polyene Phosphatidylcholine (38, 24:14)	Polyene Phosphatidylcholine Capsules (38, 26:12)	FibroScan/B-ultrasound/CT*	↓ TC, TG, ALT, AST, procalcitonin, TNF-α, IL-6, diamine oxidase, serum endotoxin, HOMA-IR regulating gut microbiota	NR	2020
Danshao Shugan Granule (88)	*Salvia miltiorrhiza* Bge. *Paeonia veitchii* Lynch *Bupleurum komarovianum* Lincz. *Curcuma aromatica* Salisb. *Cyperus rotundus* L., et al.	CHF (130); CHF + Silibinin (50)	Rosiglitazone (30), Silibinin (50)	B-ultrasound	↓ TC, TG, AST, GGT,	NR	2022
spleen-strengthening and liver-draining herbal formula (107)	*Bupleurum scorzonerifolium* Willd. *Paeonia lactiflora* Pall. *Glehnia littoralis* Fr. Schmidt ex Miq. *Atractylodes macrocephala* Koidz. *Poria cocos* (Schw.) Wolf, et al.	CHF + lifestyle (42, 27:15)	Lifestyle (40, 28:12)	Fibroscan	↓ CAP, LSM, AST, ALT; regulating intestinal flora	NR	2022
Xiaopi Huatan Granules (111)	*Bupleurum scorzonerifolium* Willd. *Artemisia capillaris* Thunb. *Atractylodes macrocephala* Koidz. *Poria cocos* (Schw.) Wolf *Alisma plantago-aquatica* L., et al.	CHF + Silibinin (60, 28:32)	Silibinin Capsules (60, 36:24)	B-ultrasound*	↓ TC, TG, ALT, AST, BMI, syndrome scores	NR	2022
Qingre Huashi Formula (110)	*Lonicera japonica* Thunb. *Polygonum cuspidatum* Sieb. et Zucc. *Coptis chinensis* Franch. *Oldenlandia diffusa* (Willd.) Roxb. *Salvia miltiorrhiza* Bge., et al.	CHF (34)	Placebo (33)	FibroScan/B-ultrasound/CT*	↓ Total symptom score, alkaline phosphatase, BMI ↑ Curative effect of TCM syndromes	NR	2022
Lingguizhugan Decoction (113)	*Poria cocos* (Schw.) Wolf *Cinnamomum cassia* Presl *Atractylodes macrocephala* Koidz. *Glycyrrhiza uralensis* Fisch.	Standard dose CHF (ITT:81, 34:47; PP:72,31:41) low dose CHF (ITT:81, 28:53; PP:71, 22:49)	Placebo (ITT:81, 34:47; PP:75, 31:44)	Liver–kidney echo ratio on ultrasound	↓ HOMA-IR, fasting insulin, HbA1c	NR	2022
Lanzhang Granules (106)	*Gynostemma pentaphyllum* (Thunb.) Makino *Astragalus membranaceus* (Fisch.) Bge. *Angelica sinensis* (Oliv.) Diels *Polygonum cuspidatum* Sieb. et Zucc. *Fritillaria thunbergii* Miq.	CHF + lifestyle (55, 30: 25)	Placebo + lifestyle (55, 35:20)	Fibrotouch	↑ HDL-C, total TCM syndrome efficacy. ↓CAP, TC, TCM syndrome score.	Slightly elevated transaminase (1)	2023

*No statistically significant; ↑ promote; ↓ inhibit. NR, no report.

### 4.1 Improving imaging findings in patients with MASLD

Several studies have found that ultrasound performed on patients with MASLD who took CHF showed a reduction in hepatic steatosis. In one RCT enrolling 260 patients with MASLD, Wang et al. showed that Danshao Shugan Granule (88) most significantly improved ultrasound finding and reduced the levels of triglyceride, total cholesterol, γ-glutamyl transpeptidase, and aspartate transaminase compared with silibinin and rosiglitazone. Another trial of Qinjiang Baoling Decoction (105) also demonstrated that the CHF had long-lasting efficacy in improving the scores of liver/spleen CT ratio and abdomen B-mode ultrasound.

Some scholars have utilized Fibrotouch to assess fatty liver grading and have found that CHF had superior therapeutic effects in MASLD. The result of one RCT showed better clinical improvement in patients with MASLD taking Lanzhang Granules (106) compared to placebo. Lanzhang Granules can effectively reduce the controlled attenuation parameter (CAP) of patients with a favorable safety profile, and significantly alleviate clinical symptoms such as right hypochondrial pain, fatigue, anorexia, and nausea. Hui et al. reached similar conclusions in their study of spleen-strengthening and liver-draining herbal formula (107), which was additionally found to reduce liver stiffness measurement (LSM) and modulate intestinal flora.

In addition, the favorable therapeutic efficacy of CHF on MASLD has been demonstrated not only by B-ultrasound but also by Fibroscan in several clinical trials. Compared with polyene phosphatidylcholine capsules, Shugan Xiaozhi Decoction (108) can better improve liver stiffness, CAP, fasting blood glucose, BMI, TG, total cholesterol (TC), aspartate aminotransferase (AST), and alanine aminotransferase (ALT). Similar conclusions were reached in the study on Qinghua Decoction by Lu et al. (109).

### 4.2 Improvement of blood tests in patients with MASLD

During the trial, some CHFs did not affect the degree of hepatic steatosis in patients with MASLD, which may be related to the relatively short intervention time. However, these CHFs improved the lipid profile and hepatic function. A multicenter, double-blind, randomized controlled clinical trial revealed that Qingre Huashi Formula (110) significantly reduced, alkaline phosphatase and body mass index (BMI) levels and improved symptoms such as bitter mouth and incomplete defecation. Long et al. found that a combination of Xiaopi Huatan Granule (111) was more effective in lowering AST, ALT, TG, TC, and BMI in patients with MASLD compared to silymarin capsules. A clinical study on Zhibitai Capsule (112) found similar results. In addition, the study also found that the treatment resulted in significant reductions in enterobacteriaceae, enterococci, staphylococcus, diamine oxidase, serum endotoxin, procalcitonin, TNF-α, IL-6, and HOMA-IR and significant increases in lactobacillus, bifidobacteria, and bacteroidetes. All the indexes of the treatment group (Zibitai capsule combined with polyene phosphatidylcholine capsule) were better than those of the control group (polyene phosphatidylcholine capsule). Besides, Lingguizhugan Decoction (113) improved insulin resistance in overweight/obese patients with MASLD by increasing the levels of DNA N6-methyladenine modification of protein phosphatase 1 regulatory subunit 3A (PPP1R3A) and autophagy related 3 (ATG3).

## 5 Conclusions and perspectives

**Figure 5:**
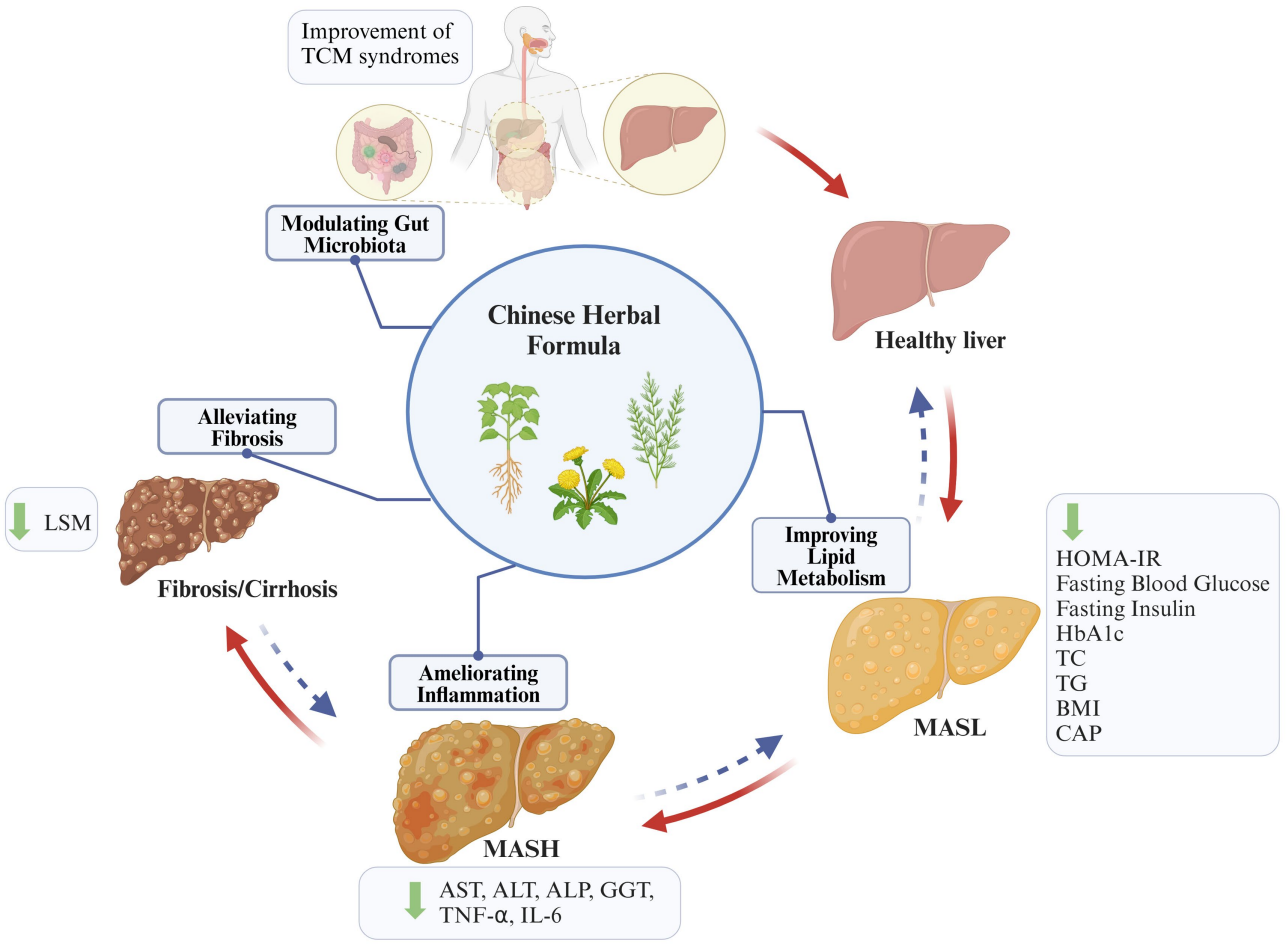
Summary of key mechanisms and events of CHF treating MASLD. CHF plays a beneficial role in inhibiting the progression of MASLD by improving lipid metabolism, ameliorating inflammation, modulating gut microbiota, and inhibiting fibrosis (created with BioRender.com).

In light of the growing prevalence of metabolic diseases, there is a heightened awareness of MASLD and a pressing need for the development of efficacious anti-MASLD drugs. However, currently, no significant therapeutic drugs with a notable curative effect are available. The pathogenesis of MASLD is complex, encompassing a sophisticated interplay of IR, abnormalities in lipid metabolism, inflammation, disturbances in gut flora, apoptosis, and fibrosis. CHFs are rich in natural ingredients and exhibit diverse biological effects in treating many diseases. Importantly, mechanistic studies and clinical trial results suggest that many CHFs have the potential to provide beneficial outcomes in the treatment of MASLD by improving lipid metabolism, inhibiting inflammatory pathways, modulating gut microbiota, and alleviating fibrosis (Figure 5). Mechanically, they primarily reverse the MASLD progression through multi-target regulation of multiple signal pathways and immune factors. In particular, NF-κB, AMPK, FASN, IL-1β, IL-6, Tnf-α, and Nrf2 are identified as key molecular targets for the improvement MASLD through CHF.

The therapeutic efficacy of CHF in MASLD has been widely explored, but toxicological studies are lacking and safety assessment has not been emphasized. To date, the results of clinical trials are limited and tend to show subtle effects compared to cellular and animal models. Furthermore, the relatively modest sample sizes and dearth of liver biopsy data in the majority of clinical studies constrain the scope for deriving robust evidence from clinical trials regarding efficacious strategies for the prevention and treatment of MASLD. The diversity of CHF sources and their chemically active components, as well as the ample anti-MASLD mechanisms, reinforce our confidence and motivation to discover new anti-MASLD drugs. Nevertheless, the screening of CHF and the associated multicenter, more rigorous, and larger-sample RCTs are imminent.

## Glossary

MASLD: metabolic dysfunction-associated steatotic liver disease
MASL: metabolic-dysfunction-associated steatotic liver
MASH: metabolic-associated steatohepatitis
CHF: Chinese Herbal Formulas
CAP: Controlled Attenuation Parameter
LSM: Liver Stiffness Measurement
PPAR: Peroxisome Proliferator-activator Receptor
FXR: Farnesoid X Receptor
TCM: Traditional Chinese Medicine
IR: Insulin Resistance
FAs: Fatty Acids
IFN-γ: Interferon-γ
KC: Kupffer Cell
LPS: Lipopolysaccharide
TLR: Toll-like Receptors
TGF: Transforming Growth Factor
HDC: Hepatic Dendritic Cell
MAPK: Mitogen-activated Protein Kinase
ASK: Apoptosis Signal-regulating Kinase
IRF: Interferon Regulatory Factors
JNK: Jun N-terminal Kinase
HFD: High Fat Diet
SD: Sprague Dawley
NF-κB: Nuclear Factor-κB
Nrf2: Nuclear Factor-erythroid 2-related Factor 2
ERK: Extracellular Signal-regulated Kinase
ER: endoplasmic reticulum
MyD88: Myeloid Differentiation Primary Response 88
RCT: Randomized Controlled Trials
NOD: Nucleotide Oligomerization Domain-like Receptors
TAK1: TGFβ-activated Kinase 1
TRAF: Tumor Necrosis Factor Receptor-associated Factor
TNFAIP3: TNFa-induced Protein 3
DKK3: Dickkopf-3
CARD6: Caspase Recruitment Domain 6
CREG: Cellular Repressor of E1A-stimulated Genes
TBK1: TANK-binding 1 Kinase 1
HSCs: Hepatic Stellate Cells
MPO: Myeloperoxidase
NR: No Report
MCD: Methionine and Choline Diet
MCDD: Methionine and Choline Deficient Diet
DSS: Dextran Sulfate Sodium
YAP1: Yes-associated Protein 1
PLIN2: Perilipin2
ACACA: Acetyl-CoA Carboxylase Alpha
NR1H4: Nuclear Receptor Subfamily 1 group H member 4
APOA1: Apolipoprotein A1
STING: Stimulator of IFN Genes
CPT1A: Carnitine Palmitoyltransferase 1α
CPT2: Carnitine palmitoyltransferase 2
CD36: Cluster of Differentiation 36
Acly: ATP Citrate Lyase
ACC: Acetyl-CoA Carboxylase
Scd-1: Stearoyl-CoA Desaturase-1
IL-1β: Interleukin-1β
Nlrp-3: Nucleotide-binding and oligo-merization domain-like Receptor family Pyrin domain-containing 3
H3K9ac: Acetyl-histone 3-lysine 9
HDAC1: Histone Deacetylase 1
IκBα: Inhibitor of κBα
HO-1: Heme Oxygenase 1
TC: Total Cholesterol
TG: Triglyceride
AST: Aspartate Transaminase
ALT: Glutamic Pyruvic Transaminase
GGT: γ-glutamyl Transpeptidase
HDL-C: High-density Lipoprotein Cholesterol
E: Liver Stiffness
BMI: Body Mass Index
HOMA-IR: Homeostasis Model Assessment of Insulin Resistance
ITT: Intention-to-treat
PP: Per-protocol
